# Discrimination of Dynamic Tactile Contact by Temporally Precise Event Sensing in Spiking Neuromorphic Networks

**DOI:** 10.3389/fnins.2017.00005

**Published:** 2017-01-31

**Authors:** Wang Wei Lee, Sunil L. Kukreja, Nitish V. Thakor

**Affiliations:** Singapore Institute for Neurotechnology (SINAPSE), National University of SingaporeSingapore, Singapore

**Keywords:** dynamic tactile sensing, neuromorphic, spatiotemporal, biomimetic, spiking

## Abstract

This paper presents a neuromorphic tactile encoding methodology that utilizes a temporally precise event-based representation of sensory signals. We introduce a novel concept where touch signals are characterized as patterns of millisecond precise binary events to denote pressure changes. This approach is amenable to a sparse signal representation and enables the extraction of relevant features from thousands of sensing elements with sub-millisecond temporal precision. We also proposed measures adopted from computational neuroscience to study the information content within the spiking representations of artificial tactile signals. Implemented on a state-of-the-art 4096 element tactile sensor array with 5.2 kHz sampling frequency, we demonstrate the classification of transient impact events while utilizing 20 times less communication bandwidth compared to frame based representations. Spiking sensor responses to a large library of contact conditions were also synthesized using finite element simulations, illustrating an 8-fold improvement in information content and a 4-fold reduction in classification latency when millisecond-precise temporal structures are available. Our research represents a significant advance, demonstrating that a neuromorphic spatiotemporal representation of touch is well suited to rapid identification of critical contact events, making it suitable for dynamic tactile sensing in robotic and prosthetic applications.

## 1. Introduction

The increased expectation of robots to be able to interact in natural dynamic environments requires the ability to quickly detect, recognize and respond to physical contact events. Such capabilities are necessary for dexterous manipulation (Johansson, 2002) and to ensure the safety of the user and robot (Lumelsky, 2005; Dahiya et al., 2013). Ideally, the entire exterior of the robot should be sensitive and responsive to touch. These requirements highlight the need for a rapid acquisition and processing mechanism to interface with thousands of tactile sensing elements distributed across the surface of a robot.

In comparison to other sensing modalities such as vision and hearing, the difficulties in acquiring and processing data from numerous tactile sensing elements, or *taxels*, have been cited as a primary reason for the limited progress in tactile sensing (Lee, 2000). The challenges are a result of taxels being spread over a large and non-uniform surface area. To minimize clutter and wiring complexity, the sampling and readout processes are often heavily time-multiplexed, leading to reduced readout data rates.

We used the human somatosensory system as our inspiration since it is unmatched in terms of scale and responsiveness compared to state-of-the-art artificial tactile sensors. With over 17,000 mechanoreceptors on the palmar side of each hand, humans demonstrate exquisite reflex, responding within 65 ms of a contact event (Johansson et al., 1992). After accounting for the multiple layers of neural processing and slow conduction velocities of axons (Buchthal and Rosenfalck, 1966), the actual window for data acquisition is estimated at ~5 ms (Johansson and Birznieks, 2004). Neurophysiologists postulate that the temporal structure of action potentials from a population of mechanoreceptors may serve as a neural code that facilitates rapid sensory processing in the brain (Johansson and Birznieks, 2004; Pruszynski and Johansson, 2014).

Among the four main classes of mechanoreceptors innervating the glabrous skin, the Meissner corpuscles respond to stimuli with the highest temporal precision at 0.8 ms (Johansson and Birznieks, 2004). Meissner corpuscles are also known as fast-adapting type 1 (FA-1) receptors since they only respond to changes in stimulus intensity and have a small receptive field. Compared to other mechanoreceptors, FA-1 afferents have a much higher innervation density of 140 afferents per cm^2^ at the fingertips (Vallbo et al., 1984). Due to large overlaping receptive fields, Meissner corpuscles are often activated as a population and in a particular order as the skin deforms upon contact. This enables the representation of contact parameters in the timing and sequence of spikes, and have been shown to encode edges, local curvature, force magnitude and direction (Johansson and Flanagan, 2009; Pruszynski and Johansson, 2014). It has been postulated that these spatiotemporal response patterns are highly compressed representations that enable rapid signal propagation and processing to occur (Johansson and Birznieks, 2004; VanRullen et al., 2005).

The utility of spatiotemporal pattern recognition is increasingly apparent in artificial tactile sensing research. Lee et al. (2013) demonstrated such an approach for discriminating between two indentation conditions and for detecting three different gait events (Lee et al., 2014). Spatiotemporal patterns were generated by detecting the time each sensor element crossed a pre-defined pressure threshold for a given stimulus. However, the use of a fixed threshold is only practical for properly calibrated arrays. Rongala et al. (2015) showed an improved accuracy for texture recognition using spatiotemporal spike patterns compared to using spike rates alone. Subsequent experiments conducted by Oddo et al. (2016) demonstrated that the temporal structure of signals used in peripheral nerve stimulation enhanced the ability of human subjects to discriminate textural features. While all the papers cited above have commented on the importance of precise spike timing, the temporal precision of the representations used were not quantified in detail. A thorough study that establishes the temporal resolution required to maximize the efficacy of spatiotemporal representations will be highly relevant to the field.

Firstly, we propose the use of spatiotemporal representations of tactile pressure changes to resolve dynamic contact events that are common during daily interactions with our environment. Secondly, we demonstrate the feasibility of our approach and investigate the optimal temporal precision necessary to capture maximum information for classification of contact events. Thirdly, we demonstrate how an event-based representation allows tactile signals of high temporal fidelity to be preserved while maintaining a reasonable communication bandwidth.

The paper is organized as follows. In Section 2 we give an overview of the state-of-the-art tactile sensor arrays as well as alternative approaches to improve sampling rates. Subsequently in Section 3 we describe the methods used in our study, including both the finite-element-model (FEM) and physical experiments applied in the study. Our results are presented in Section 4. In Section 5 we provide a discussion about our findings and concluding remarks about this study.

## 2. Background

The ability to rapidly sample large arrays of tactile elements spaced across a non-uniform surface area remains a major challenge. While multiplexing of wires is necessary to reduce the mechanical complexity of the system, the total communication bandwidth of the sensors is reduced as a consequence. Currently, the fastest tactile sensor arrays can communicate at 1.9 kHz but with only 256 sensing elements (Schürmann et al., 2012), which is insufficient to cover the body of a reasonably sized robot (see Table 1). Limited communication bandwidth has been cited as a restriction on the number of sensing elements on an array. Although future developments of high speed communication protocols will increase the sampling rate of tactile sensors, it is unclear whether such a large volume of information is necessary. Increased data acquisition requires more complex algorithms and hardware, which leads to higher implementation costs and power consumption. A practical approach is to intelligently reduce the data generated from the sensing front-end, prior to transmission.

**Table 1:** **Comparison of the human hand to tactile sensor arrays of comparable scale/speed**.

**Sensor**	**Number of taxels**	**Sampling rate (Hz)**	**Output format**	**References**
Gifu Hand III	859	100	8 bit pressure	Mouri et al., 2002
Slip detection array	256	1900	12 bit pressure	Schürmann et al., 2012
FPGA data compression	640	200	Fitted ellipses of pressure distribution	Oballe-Peinado et al., 2012
CellulARSkin network	780	250	Events with 12 bit pressure	Bergner et al., 2015
TactArray system (Pressure Profile Systems)	1024	10	12 bit pressure	Pressure Profile Systems, 2016
Industrial I-scan system (TekScan)	1936	100	8 bit pressure	TekScan, 2016
Human hand	~17,000	>1000 (equivalent)	Action potentials	Johansson and Flanagan, 2009
Our system	4096	5200	Events with 1 bit pressure change	Lee et al., 2015

*Note that mechanoreceptors are not frame-based but asynchronous. Due to their sub-millisecond temporal precision, they could achieve an equivalent sampling rate of >1000 Hz*.

Spatial resolution is often compromised to improve response times. Shimojo et al. (2010) developed a sensor optimized to only detect the centroid and magnitude of an applied load. This enables rapid detection of contact (1 ms) but leads to severely compressed spatial resolution of a single point. Oballe et al.'s implementation compressed the tactile image into an ellipse to represent location, distribution and magnitude of a load (Oballe-Peinado et al., 2012). This work is similar to Shimojo et. al. since there is only enough resolution to represent a single point of contact. The technique employed by Fukui et al. (2011) selectively sampled the sensor array based on a genetic algorithm, increasing the probability of detecting a contact event without increasing the sampling rate. However, this approach is ideally suited for localized contacts since scanning a large area is still slow due to a limited acquisition speed of 0.2 ms per taxel. Similarly, the sensor array presented by Schmitz et al. (2011) can only achieve a higher sampling rate of 500 Hz by averaging all sensing points. Although these techniques permit rapid detection of contact events, reduced spatial resolution does not provide accurate estimates of key contact properties such as force magnitude, direction and object curvature (Johansson et al., 1992; Johansson and Birznieks, 2004). Precise estimates of these characteristics are critical for sensory control during object-oriented actions (Johansson and Flanagan, 2009).

An implementation by Bergner et al. (2015) used an asynchronous communication network to propagate pressure measurements. Packets containing pressure intensity and other measurements were sent only when a significant change in pressure was detected at individual sensing modules. Due to the sparse nature of tactile signals, information from 780 taxels sampled at 250 Hz were reliably transmitted while consuming less than half the communication bandwidth under a worse case scenario. While the results were promising, the focus of the work was to reconstruct 2D pressure maps with minimal error. We believe this application and performance is complementary to the spatiotemporal representation proposed in this paper.

## 3. Methods

This section describes the two main methods used in the investigation of our hypothesis—FEM modeling and physical experiments. The section begins with a discussion on the origins of spatiotemporal features in mechanoreceptor responses and how the representations are reproduced using our FEM model. This is followed by a description of the physical experimental setup. Finally, we present the methodology used to compute the information content within outputs of both the FEM simulation and recordings from the physical sensor array.

### 3.1. Monte Carlo FEM simulation

**Figure 1:**
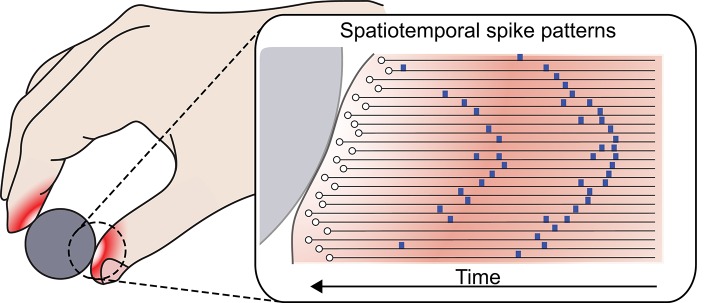
**Formation of spatiotemporal spike patterns**. Skin deformation during mechanical contact produces distributed patterns of pressure that triggers tactile afferents in a sequence to produce a spiking response pattern.

During mechanical contact, skin deformation results in distributed patterns of pressure that trigger tactile afferents in a precise sequence, creating spike patterns that are spatially and temporally distinct (Figure 1). The number of unique representations or dimensionality of a spatiotemporal code is dependent on the (a) length of decoding time window, (b) temporal precision of the spikes, and (c) number of mechanoreceptors involved. Experimentally, it has been observed that human fingertips are innervated with an exceptional density of mechanoreceptors (Johansson and Flanagan, 2009). The afferents exhibit highly overlapping receptive fields, partly as a consequence of the soft and conformable supportive tissues beneath (Johansson and Flanagan, 2009). Combined with sub-millisecond temporal precision, spatiotemporal spiking patterns have the potential to encode a large range of stimuli within a short time window. Using this biological mechanism as an inspiration, we designed our simulations and experiments to target soft sensors with high density.

**Figure 2:**
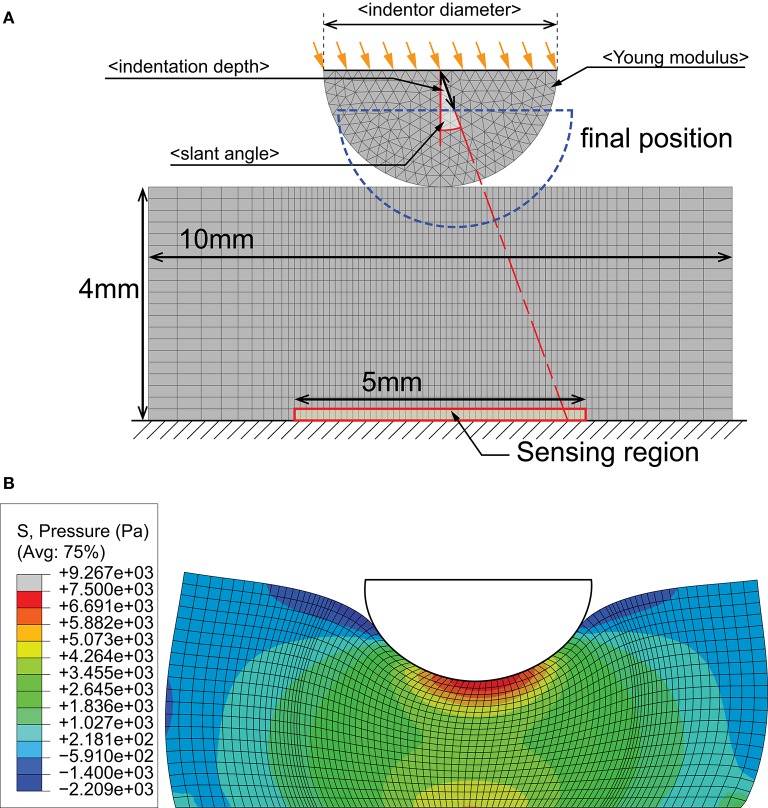
**FEA simulation of indentation by an edge represented by a half-circle. (A)** Variables in the simulation include indentor diameter, indentor hardness (Young modulus), depth of indentation and angle of indentation. **(B)** Example output from an indentation at 10 deg slant. Colored contours represent pressure distributions.

We restricted this study to transient tactile events that can occur on a generic 5 × 5 mm patch of surface to ensure the problem remains computationally tractable. Since the two-point discrimination threshold of human fingertips is between 2 and 4 mm (Johnson and Phillips, 1981), it is reasonable to assume that we only have to resolve a single edge feature within this region under most interaction scenarios. Moreover, the sensor surface is assumed to be flat due to the small region of interest. This reduces the problem to one of analyzing object indentations, where an edge is depressed onto a flat surface. Taking advantage of symmetry properties to reduce computational complexity, the model was constructed in 2D. Figure 2 illustrates the simulation framework.

To faithfully and accurately synthesize interaction scenarios, we selected several contact parameters to vary (see Table 2). The indentor was modeled to have linear elastic properties, while the sensor was modeled using the parameters listed in Table 3. These conditions accurately simulate Dragon Skin® 10 (Smooth-on, USA) silicone rubber commonly used as a cover for soft tactile sensors (Elango and Faudzi, 2015).

**Table 2:** **Table of experimental variables**.

**Variable**	**Values (increment)**	**Unit**
Indentor diameter	2–4 (0.5)	mm
Young modulus of indentor	50–250 (100)	kPa
Indentation depth	0.5–2 (0.5)	mm
Indentation angle	0–20 (5)	degrees

**Table 3:** **Sensor material constants (3 term Yeoh hyperelastic model)**.

**Material type**	***C*****_10_ (Pa)**	***C*****_20_ (Pa)**	***C*****_30_ (Pa)**	**SSE**
Dragon Skin 10®	3600	25.8	−0.056	0.12

**Figure 3:**
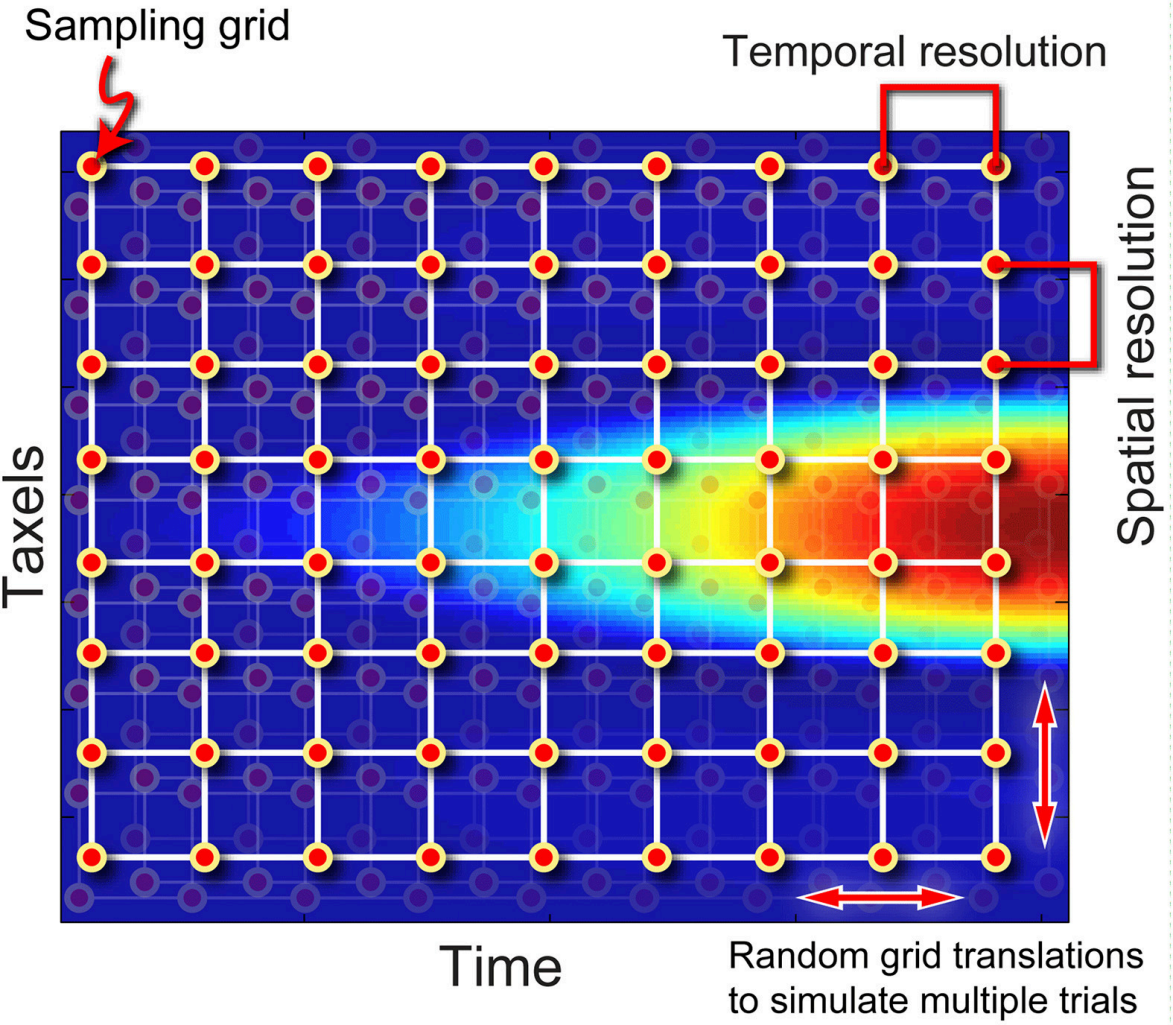
**Illustration on the sub-sampling procedure**. By randomly jittering the sampling grid, multiple variations can be generated from the same FEA data, simulating experimental error due to limited spatiotemporal resolution of a physical sensor.

For a given parameter set the result from a FEM simulation is deterministic. To assess the robustness of our approach we simulated stochastic variations using a sub-sampling technique. A sub-sampling grid was constructed where the horizontal and vertical spacing of sample points corresponded to the temporal and spatial resolution of the simulated sensor array, respectively (Figure 3). Each sub-sampled output was generated by translating the sub-sampling grid within the FEM output, thus ensuring that while individual sampling points are spaced in accordance to the sampling period and spatial resolution of the simulated sensor array, the absolute time and location of the sampling points are different with each variation. The grid translations in the temporal and spatial axes were uniformly random in nature to create outputs that are stochastic, analogous to the uncertainty involved due to limited sensor resolutions. Using this technique, a change in simulated sampling period corresponds to a change in the density of the sub-sampling grid along the time axis.

The FEA experiments were conducted on ABAQUS 6.13-1. The contact event was the indentation of a half-circle onto a rectangular substrate at a constant velocity (Figure 2). Displacement control was used, with full displacement achieved in 100 ms. The friction coefficient between the indentor and substrate was fixed at 0.7. All outputs were recorded at 0.2 ms time steps and 0.1 mm spatial resolution, from a sensing region of 5 mm width. Data from the base sensing region was recorded and sub-sampled to 25 elements to be compatible with the density of mechanoreceptors (Johansson and Vallbo, 1979). The spline interpolation technique in MATLAB (MathWorks, MA, USA) was used to estimate values between discrete points of an FEM output. Five-hundred variations were generated per experimental condition, thus resulting in a dataset of 150,000 samples.

#### 3.1.1. Simulation of mechanoreceptor transduction

Pressure was used as the input parameter to the computational model since it well describes the transduction of tactile sensors (Cabibihan et al., 2014). We extracted pressure estimates from elements of the same depth within the sensing region and mapped them to an exponential scale. The exponential mapping approximates the response curves of mechanoreceptors (Johansson et al., 1980) as well as piezoresistive transducers commonly used in artificial tactile sensors (Paredes-Madrid et al., 2011). The exponential conversion is described as:

(1) $$\begin{array}{l} \rho_{n}\left(t\right)={\left(u_{n}\left(t\right)\right)}^{\alpha } \end{array}$$

where *u*_*n*_(*t*) is the pressure experienced by sensor element indexed *n* at time *t*, while α is a fixed power coefficient.

To analyze the information captured by spatiotemporal spike representation, a conversion from analog pressure signals to spikes is needed. This transformation was achieved using a phenomenological FA-1 afferent model. In most transduction models of FA-1 mechanoreceptors, the first derivative of pressure is the primary input component to a leaky integrate-and-fire neuron model (Bensmaia, 2002; Kim et al., 2010). Therefore, we defined the input current *I*_*n*_(*t*) to the neuron model corresponding to element *n* at each time instance *t* as the change in pressure:

(2) $$\begin{array}{l} I_{n}\left(t\right)=\kappa \left(\frac{d\left(\rho_{n}\left(t\right)\right)}{dt}\right) \end{array}$$

where ρ_*n*_(*t*) is pressure at time *t* after accounting for the exponential relation (Equation 1), and κ = 1 is a unit constant symbolizing the transduction from pressure to neural input current. Neuron dynamics are described by its membrane potential *U*_*n*_(*t*), which is approximated by the integration the input currents since the last spike *t*_s*pk*_:

(3) $$\begin{array}{l} \frac{d\left(U_{n}\left(t\right)\right)}{dt}=\frac{1}{C}\overset{t}{\underset{t=t_{\text{s}pk}}{\sum }}I_{n}\left(t\right). \end{array}$$

where *C* is the membrane capacitance of the neuron model. A spike is discharged when the membrane potential exceeds a threshold θ_*n*_(*t*). In this work, we introduce polarity to the output spikes, with:

(4) $$\begin{array}{l} s_{n}\left(t\right)=\left\{ \begin{array}{ll} 1\; & \text{if }U_{n}\left(t\right)>\theta_{n}\left(t\right)\\ -1\; & \text{if }U_{n}\left(t\right)<-\theta_{n}\left(t\right)\\ 0\; & \text{otherwise.} \end{array}\right. \end{array}$$

**Figure 4:**
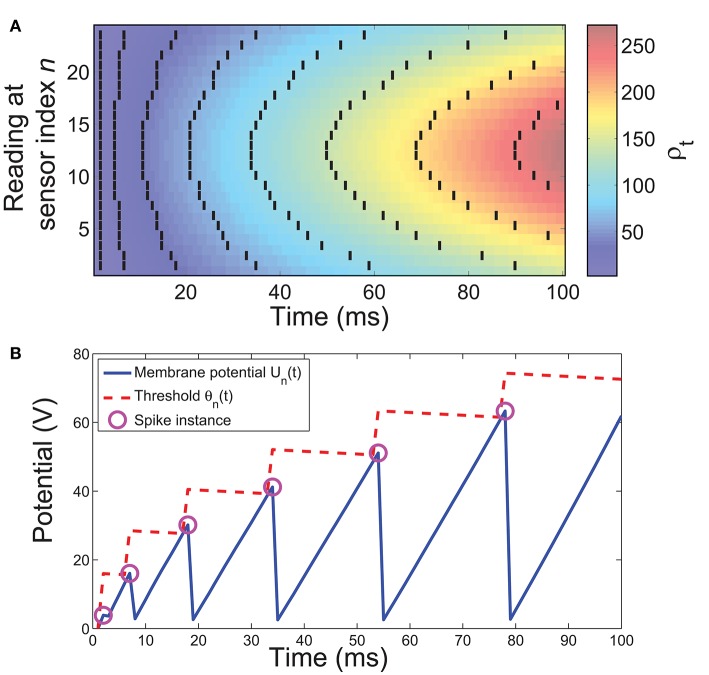
**Illustration of the spike conversion process. (A)** A composite illustration of spikes obtained using simulated data. The background color gradient represents pressure values (ρ_*t*_) after accounting for exponential relation. Solid black lines represent the spikes discharged by our model. **(B)** The dynamics of an example conversion neuron. A spike is discharged when the membrane potential *U*_*n*_(*t*) exceeds the threshold θ_*n*_(*t*). Immediately after a spike, *U*_*n*_(*t*) is reset to 0 while θ_*n*_(*t*) is increased, θ_*n*_(*t*) decays toward baseline otherwise.

A negative spike representation is useful since a decrease of pressure may be experienced at the periphery of the sensor, especially when the indentation is performed at larger slant angles. Upon spike emittance, *U*_*n*_(*t*) is reset to 0 mV and the threshold is incremented. This property increases the difficulty of a consecutive spike and simulates a refractory period of the mechanoreceptor. The increased threshold slowly decays to a baseline value θ_0_ when there are no spiking activities. The dynamics of the threshold is described as:

(5) $$\begin{array}{l} \theta_{n}\left(t\right)=\left\{ \begin{array}{ll} \theta_{n}\left(t-1\right)+TA\theta_{0}\; & \text{after a spike}\\ \theta_{n}\left(t-1\right)-B\theta_{0}\; & \text{if }\theta_{n}\left(t-1\right)>\theta_{0}\\ \theta_{0}\; & \text{otherwise} \end{array}\right. \end{array}$$

where *A* and *B* are constants that control the rate at which the threshold potentiation and decay occur respectively and *T* is the sampling period of the system in milliseconds. The dynamics of the model are illustrated in Figure 4B while Figure 4A shows how the corresponding spatiotemporal pattern is obtained from a single sample.

#### 3.1.2. Analysis for simulated data

Information theory is widely used in neural systems to understand the statistical details embedded in spike trains. The information encoded in neural recordings are measured by the ability to discriminate various stimuli based on their spike train response. We used linear discriminant analysis (LDA) to classify the outputs, where inputs to the classifier are spike raster plots of size *m* × *n*, representing outputs from *m* mechanoreceptors lasting *n* time steps (where *n* = 100/sampling period (ms)). Shannon's information measure (Shannon, 2001) was applied to compute the mutual information *I*(*R*; *L*) between the ground truth label (*L*), and classifier response (*R*):

(6) $$\begin{array}{l} I\left(R;L\right)=\underset{R,L}{\sum }p\left(R|L\right)\cdot p\left(L\right)\log_{2}\frac{p\left(R|L\right)}{p\left(R\right)} \end{array}$$

where *p*(*R*|*L*) is the probability of the classifier emitting response *R* for an input *L*, as derived from the confusion matrix. This approach presents a more objective performance measure since it provides information about the error distribution (non-diagonal values in the confusion matrix), not just the number of hits (diagonal values). Results were computed using the information breakdown toolbox (Magri et al., 2009) implementing the Panzeri-Treves method (Panzeri and Treves, 1996) for bias correction.

For all experiments, the constants described in Table 4 were used. The value of θ_0_ was estimated by dividing the range of pressures by 1024, the smallest pressure change that can be resolved by a 10 bit ADC. Values for the exponential constant α of biological mechanoreceptors have been reported to range between 0.2 and 1.4 with a mean of 0.7 (Vallbo et al., 1984). For computational simplicity, the membrane capacitance *C* of the integrate and fire neuron model was fixed at 1. The values for *A* and *B* were obtained heuristically to ensure that the model's spike rate is biologically plausible at around 100 Hz.

**Table 4:** **List of constants used for simulation**.

**Constant**	**Symbol**	**Value**
Exponential constant	α	0.7
Baseline threshold	θ_0_	3
Threshold potentiation	A	20
Threshold decay	B	0.05
Membrane capacitance	C	1
Trials per simulation	K	500

### 3.2. Physical experiment

While simulating results using FEM is useful for controlled variation of contact parameters, it is also important to be able to validate the findings observed in simulation through physical experiments. In this section, we provide details for a set of physical experiments to assess the predictions from FEM simulations.

#### 3.2.1. The kilotaxel-kilohertz tactile sensor array

**Figure 5:**
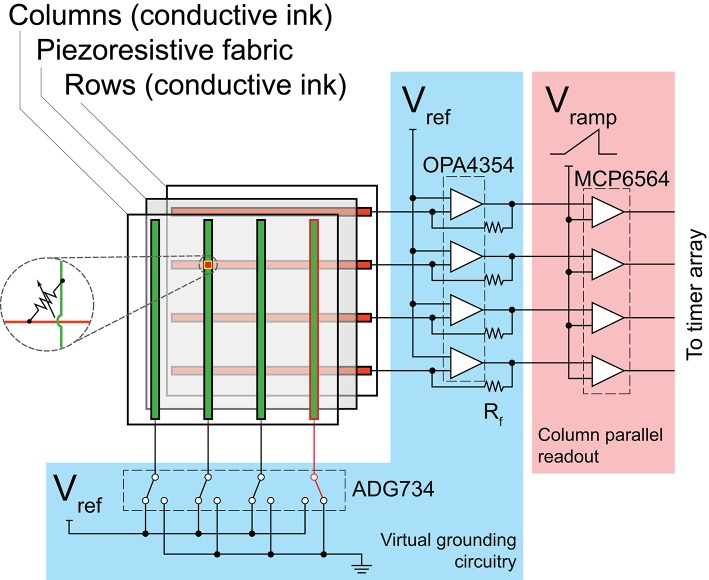
**Sensor architecture and readout circuitry**. Blue region: circuitry for cross-talk cancellation. Red region: Circuitry for analog to digital conversion.

We previously reported a high speed tactile sensing platform to investigate the use of spatiotemporal coding in touch (Lee et al., 2015). It has 4096 piezoresistive sensing elements arranged in a 64 × 64 grid. Using a row-parallel column readout technique a very high sampling rate of 5.2 kHz was achieved. The sensor elements were made by inkjet printing of conductive traces as rows and columns on separate sheets of polyethylene (AgIC Inc., Tokyo, Japan). The traces were then arranged orthogonally, with a piezoresistive fabric (LR-SLPA, Eeonyx, CA) sandwiched in between, where each intersection formed a sensing element (Figure 5). The size of each element measured 2.29 × 2.29 mm and the entire active region covered an area of 17 × 17 cm. The sensor array was covered by a 10 mm layer of polyurethane foam to provide the mechanical compliance needed to capture spatiotemporal patterns.

Sampling was controlled by a field-programmable-gate-array (FPGA) from Xilinx (XC6SLX45). A/D conversion was performed using a single-slope comparator array and multiple parallel timers (Figure 5). The FPGA was programmed to produce a spike event whenever a significant change in pressure (≻ 0.02 N) was detected. Each output event indicates the time, coordinates, and the magnitude of the pressure change. Notice that while all elements of the sensor array are sampled in each frame, the output from the FPGA is event-based with the temporal fidelity matching that of biological mechanoreceptors.

#### 3.2.2. Experimental setup

**Figure 6:**
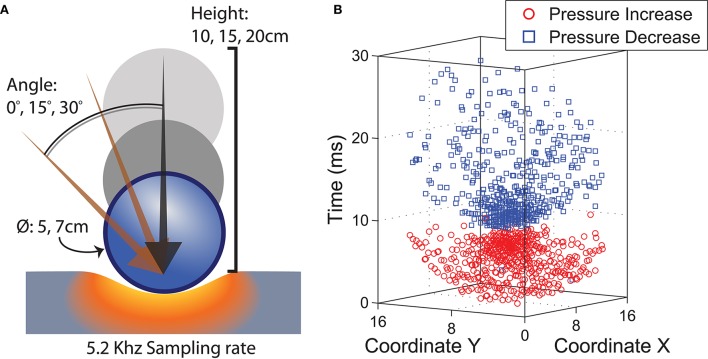
**(A)** Ball dropped on sensor substrate under various physical conditions. **(B)** Spatiotemporal events captured by sensor array.

Similar to the FEM simulations, we performed a series of indentation tests on the sensor array. Rigid 3D printed spheres of size 5 and 7 cm in diameter were released from heights of 10, 15, and 20 cm vertically above the sensing surface. The experiments were repeated with the sensor at a slant of 0, 15, and 30 degrees, resulting in a total of 18 different combinations of contact parameters. Figure 6A provides a graphical illustration of the various experimental scenarios. For each parameter combination 100 trials were performed. The weight of all spheres was calibrated to be similar (36 ± 1 g).

We extracted a region of interest consisting of 16 × 16 elements centered around the averaged spatial coordinates of the elements that responded during the first sampling period upon impact. The duration of each recording was standardized to 50 ms, which is long enough to capture the first impact but not subsequent bounces. Figure 6B encapsulates data recorded from a representative impact stimulus from a 5 cm sphere dropped from a height of 10 cm with a 0° slant angle. Data corresponding to increased sampling periods were generated by quantizing the timestamps of the events into bins of lower temporal precision.

#### 3.2.3. Analysis for experimental data

Sampling the sensors at a high rate yields a sparse output signal with high dimensionality. Classifying the spike raster plots using LDA is computationally intensive and, hence, impractical. Therefore, we adopted a metric space approach commonly applied in neuroscience to compute mutual information (Victor and Purpura, 1997). Pairwise distances between trials were first computed using a modified Van Rossum distance metric (van Rossum, 2001). Based on the calculated distances, a K-nearest neighbor clustering technique was implemented to classify the data and mutual information was computed from the classification results (see Equation 6).

The Van Rossum distance metric was originally developed to measure the difference of two spike trains and can be summarized as follows (van Rossum, 2001). Each spike is convolved with an exponential kernel to obtain a continuous signal:

(7) $$\begin{array}{r} f\left(t\right)=\overset{I}{\underset{i=1}{\sum }}H\left(t-t_{i}\right)\text{ exp }\left(-\frac{t-t_{i}}{\tau }\right) \end{array}$$

where:

(8) $$\begin{array}{rc} H\left(x\right) & =(\begin{array}{l} 0\text{ if }x<0\;\\ 1\text{ if }x\geq 0\; \end{array} \end{array}$$

and *t* ∈ ℝ^+^ is time, *t*_*i*_ is the time of the *i*th spike, *H*(·) is the Heaviside step function and τ is a free parameter that affects temporal smoothing of the signal. The distance metric is defined as the Euclidean norm on the function space:

(9) $$\|f_{\left(a\right)},f_{\left(b\right)}{\|}_{2}=\sqrt{\sum_{t=0}^{T}(f_{\left(a\right)}(t)-f_{\left(b\right)}(t){)}^{2}}.$$

In this work we extended the computation to patterns involving multiple spike trains by applying a spatial kernel to the continuous signal *f*(*t*). The spatial smoothing procedure for spike train indexed *m* for a spatiotemporal pattern with *N* spike trains is expressed as:

(10) $$\begin{array}{l} g_{m}\left(t\right)=f_{m}\left(t\right)+\overset{N}{\underset{n=1}{\sum }}\left(f_{n}\left(t\right)\cdot \text{exp}\left(\frac{-d_{m,n}}{\sigma }\right)\right) \end{array}$$

where *d*_*m,n*_ is the physical distance between sensor elements indexed *m* and *n* and σ is the spatial smoothing parameter.

The distance between two spatiotemporal patterns indexed *a* and *b* with *N* spike trains is calculated as:

(11) $$\|g_{\left(a\right)},\;g_{\left(b\right)}{\|}_{2}=\sqrt{\sum_{n=1}^{N}(g_{n(a)}(t)-g_{n(b)}(t){)}^{2}}.$$

In this work, we used τ = 0.4 ms and σ = 2 mm because they yield the highest mutual information after a grid search of τ ∈ [0.1, 2] ms at intervals of 0.1 ms and σ ∈ [1, 5] mm at intervals of 1 mm.

## 4. Results

This section presents our findings from the FEM simulations and physical experiments, with an emphasis on how information may be lost due to reduced temporal resolution.

### 4.1. Information embedded in spatiotemporal features

**Figure 7:**
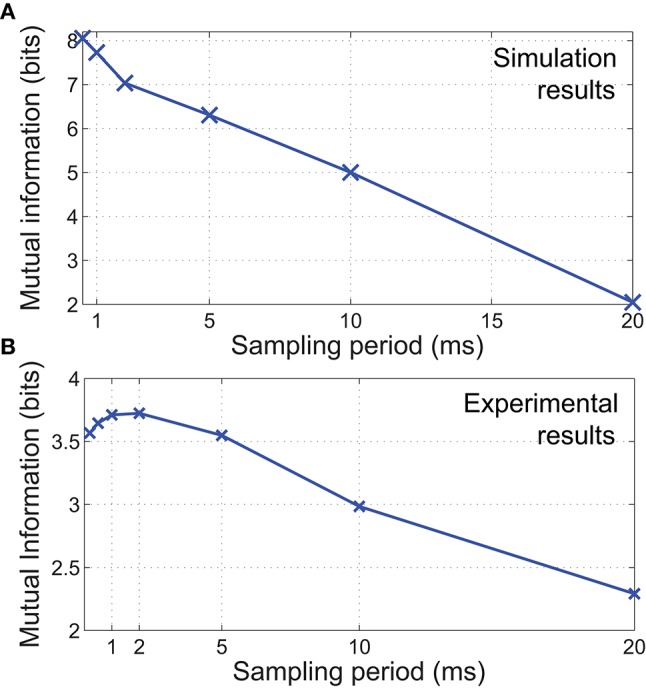
**In both (A)** simulated and **(B)** experimental scenarios, mutual information is observed to decrease when sampling periods are increased, resulting in a reduction of stimulus discriminability. We also observed a decrease in mutual information for sampling periods <1 ms for physical experiments. This might be caused by increased high frequency noise when sampling at faster rates.

The synthetic data was classified based on the combination of stimulus parameters (total of 300 classes). The results in Figure 7 illustrate that mutual information decreases significantly along with the sampling rate. Although the duration of stimulus presentation is significantly longer at 100 ms, we observed a large discrepancy in mutual information between sampling with 0.5 and 20 ms periods. This difference in performance indicates that while a sampling period of 20 ms is within Nyquist limits for detecting the indentation event, the spatiotemporal features needed for fine discrimination is contained at time scales notably shorter than the duration of a contact event.

Results from the classification of physical data showed that maximum information was obtained when sampling at 1 ms periods (Figure 7). In agreement with the FEM simulations, mutual information generally decreases with sampling rate. However, a sampling period below 1 ms also led to a decrease in mutual information. This may be due to increased high frequency noise when sampling at faster rates, resulting in a reduction of spatiotemporal pattern consistency.

### 4.2. Discriminability of individual contact parameters

In a realistic setting, it is often necessary to distinguish stimuli based on a single parameter, regardless of how other properties may have changed. For instance, the hardness of objects may need to be identified regardless of its shape. Here we examine how the reduction in temporal precision affects the discrimination of individual contact parameters.

**Figure 8:**
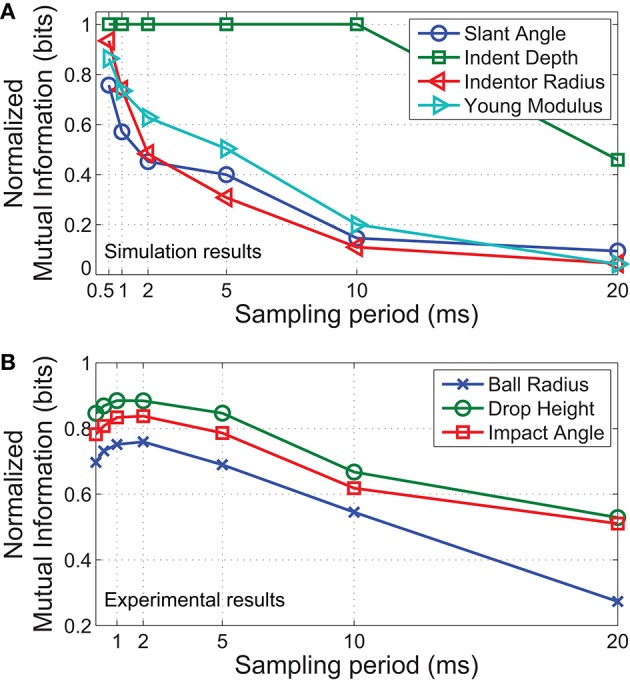
**Normalized mutual information vs. sampling period for each parameter varied. (A)** Results from simulated contact. **(B)** Results from physical experiments. In both cases, the radius of the indentor is most significantly affected by a decrease in temporal precision, while indentation depth/drop height is the least affected parameter. Note the logarithmic scale on the x axis.

For simulated data, we partition all 150,000 samples into 4 classes when classifying based on slant angle/indentation depth/indentor diameter or 3 classes when classifying for hardness (Young modulus). For physical recordings, all 1800 trials were split into 3 classes for slant angle/drop height or 2 classes for ball radius. Results from both the simulations and physical experiments were normalized to the maximum mutual information (log_2_(# of classes)) for comparison. Figure 8 illustrates the results of this study. As observed earlier, mutual information decreased with sampling period for most parameters. However, the effects of a decreased sampling period are most noticeable for discriminating stimuli of different indentor radii (Figure 8). In contrast, differences in indentation depth do not require a high sampling rate for robust discrimination, possibly because good discriminability can already be achieved based solely on the number of spikes elicited.

In concurrence with our FEM simulations, results from physical experiments show that discriminability of the ball radius is the most affected parameter as sampling period increases (Figure 8). Since the extracted region of interest is less than the full contact area, the size of the ball can only be distinguished based on small differences in spike times, an effect of variations in local curvature.

### 4.3. Classification latency

Accurate classification is beneficial but has little advantage if higher performance is achievable only after the entire stimulus is recorded. Here we process samples truncated in time to investigate information content with respect to the duration of stimulus recorded.

**Figure 9:**
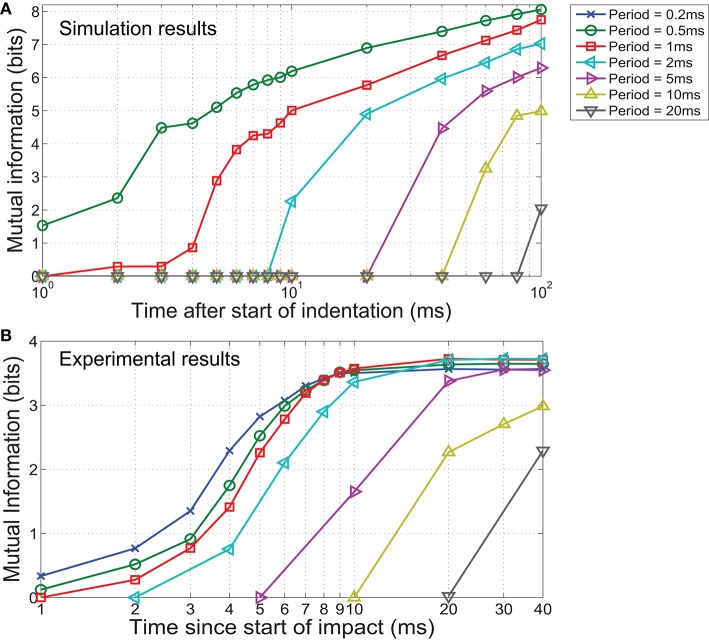
**Mutual information as a function of sampling period during stimulus presentation**. In both the **(A)** simulated and **(B)** experimental scenarios, mutual information increases earlier for a shorter sampling period (higher temporal resolution) and reached a higher value at the end of the stimulus, indicating that better classification is achievable in less time when higher temporal resolution is available. Note the logarithmic scale on the x axis.

From our analysis on simulated data depicted in Figure 9, it is observed that additional information at fine temporal scales exists at the early stages of indentation. For example, over 6 bits of mutual information is available in a 10 ms recording when sampling with a period of 0.5 ms, while similar performance can only be achieved in an 80 ms recording if a sampling period of 5 ms was used. Similarly for physical trials, mutual information almost plateaued within the first 10 ms of the impact when a sampling period less than or equal to 2 ms was used (Figure 9). Slower sampling rates took at least twice as long to achieve comparable performance.

This feature is an important advantage when working with event-driven sensory control algorithms, since it has the potential to enable critical contact events to be identified quickly (Johansson and Cole, 1992).

### 4.4. Effects of increasing pressure resolution

While decreasing the temporal precision has been shown to lower mutual information, the additional communication bandwidth afforded by a lower sampling rate would allow each sample to encode pressure information in greater detail. It is thus necessary to investigate the optimal trade off between sampling rate and pressure information encoded, given a fixed readout data rate.

We modified the FA-1 model in Equation (4), allowing for spikes that also indicate the magnitude of pressure change. The dynamics of the updated model $\tilde{s}\left(t\right)$ are described as a quantization of *U*(*t*) scaled by θ_0_:

(12) $$\begin{array}{l} \tilde{s}\left(t\right)=\left\lfloor \frac{U\left(t\right)}{\theta_{0}}\right\rfloor . \end{array}$$

where ⌊·⌋ is the floor function. Modifying the output as given in Equation (12) preserves information about magnitude of pressure changes while maintaining the spiking nature of the output. Depending on the number of output bits available, $\tilde{s}\left(t\right)$ may saturate at 2^bits^ − 1. Note that only data from the FEM simulations were used for this analysis, as the output from the physical sensor has only a single bit per event.

**Figure 10:**
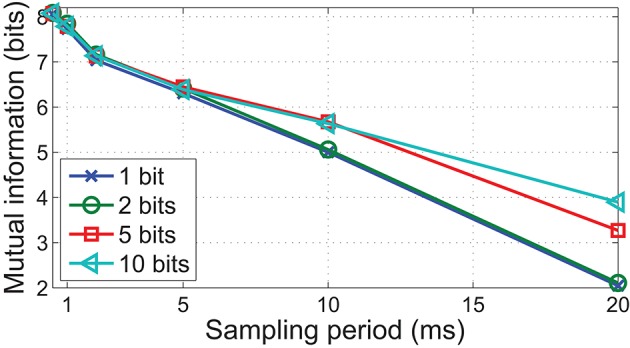
**Mutual information as a function of pressure resolution and sampling period, using data from FEM simulations**. Note how a higher bits-per-sample improves mutual information only when sampling periods are large.

The results exhibited in Figure 10 illustrate that increasing pressure resolution leads to higher mutual information. This effect is most discernible at lower sampling rates where changes in pressure intensity between consecutive samples are more abrupt. However, the improvement is less significant at higher temporal fidelity. For example, sampling with a period of 20 ms with 10 bits of pressure resolution achieves a much lower performance compared to sampling at 2 ms period with 1 bit pressure resolution, despite producing data at the same rate. These results demonstrate that at least for dynamic edge indentation stimuli, key features for discrimination are pre-dominantly of much higher frequency than the contact duration suggests, and it is thus prudent to allocate more resources to encode temporal dynamics than pressure intensity.

### 4.5. Comparison of bandwidth utilization

Sampling rates of 1 kHz and above are not practical when interfacing with thousands of sensor elements using conventional frame-based representations. Here, we describe how a bio-inspired event-based representation can reduce the amount of data generated, allowing for rapid sampling rates needed to capture spatiotemporal features. Therefore, we present a comparison of three encoding strategies.

1. Event-based change coding (EC): Event packets are generated only when a significant difference in pressure is detected. Each event packet consists of 9 bits, with 8 bits to encode the address of the element and 1 bit to indicate an increase or decrease in pressure.
2. Frame-based intensity code (FI): Pressure at each element is represented with *n* bits per sample. For this analysis, we used *n*=10.
3. Frame-based change code (FC): Only 2 bits were used per element to indicate whether an increase, decrease, or no change in pressure was observed.

Note that the 8 bit address space for the EC method was selected because it is the minimum number of bits required to encode a 16 × 16 element region of interest. Although a larger number of sensor elements require more bits to address, it is also likely more sparse because of a lower likelihood of activating all elements at once. The duration of an impact was determined as the time between the first and last event recorded during contact. Data rates for frame-based (FI and FC) approaches were computed at 5.2 kHz frame rates to match the temporal resolution of the event-based (EC) approach.

**Figure 11:**
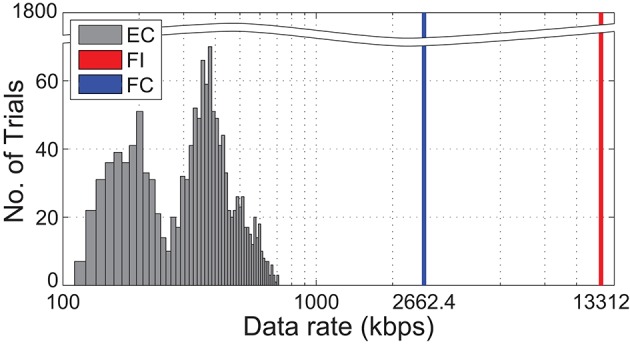
**Comparison of output data-rate among the 3 coding strategies based on all 1800 physical trials**. Event-based change (EC) code is activity dependent, hence its distributed nature. Both frame-based change code and intensity code (FC and FI, respectively) produce data at a constant rate for all trials. Note the broken y-axis and logarithmic scale on the x-axis.

Figure 11 illustrates the distribution of data-rates for all recorded impacts. An event-based (EC) code produces data at a rate that is dependent on activity level. With an EC approach, only a maximum of 659 kilo-bits-per-second (kbps) was required to fully capture the spatiotemporal events. In contrast, the frame-based codes (FI and FC) required a constant 13.3 and 2.66 Mbps respectively.

## 5. Discussion

Neurophysiologists have emphasized the benefits of spatiotemporal spike patterns in tactile processing (Johansson and Birznieks, 2004; Harvey et al., 2013), although few sensor and robotic system developers have taken this approach. The lack of adoption by the robotics community is not due to an absence of enthusiasm but because tactile sensors have traditionally been designed for accurate pressure measurements at specific points (Dahiya et al., 2013). A paradigm shift in the method of sensory acquisition and processing may be needed to utilize temporal structures efficiently.

Our work in this paper focused on precise detection of spatial and temporal features of pressure changes across a large population of taxels. Through FEM analysis and physical experiments, spatiotemporal patterns of pressure changes were used to discriminate contact scenarios involving multiple combinations of stimulus parameters such as local curvature, indentation magnitude, angle and indentor hardness. We observed that signals with temporal resolution much higher than the duration of contact were needed to resolve the various contact parameters. Loss of information from reduced sampling rates could not be recovered by increasing pressure resolution. It is most probable that the key features for discrimination of dynamic contact are pre-dominantly high frequency, and our physical experiments suggests that a tactile sensing system with sampling rates of 500 Hz and above is needed to fully exploit these features.

Although event-based communication protocols have been advocated in earlier work as a method for data reduction (Bergner et al., 2015; Rongala et al., 2015), our composition extends beyond prior literature by elucidating the increased information capacity of a spatiotemporal representation, if the events are temporally accurate. By applying the concept to our high speed sensor array, we demonstrated the ability to rapidly sample thousands of sensing elements while preserving the structure of response patterns with sub-millisecond precision needed for accurate classification. We also observed an approximately 20 time reduction in data transmitted during ball-drop experiments when compared to conventional frame-based representations. The savings incurred for high density sensors covering large surface areas would likely be much higher using event-based coding since it is unlikely that changes will be detected for all elements at every sampling period.

Realistic tactile stimuli are highly complex. We do not contend that our simulations and experiments have comparable diversity. We restricted our investigation to transient dynamic stimuli since these are some of the most challenging problems in tactile sensing. In addition, for robots to operate safely in natural dynamic environments, the ability to react quickly to complex transient events is crucial.

A comparison of the results obtained from physical experiments to FEM simulations reveals less than predicted improvement in performance when sampling with periods below 2 ms (Figure 7). This might be a consequence of stochastic error introduced by nature of the experimental trials. Sources of inconsistencies include the varying response characteristics of sensing elements and manual stimuli delivery. Both introduce large intra-class variations in the spatiotemporal patterns in comparison to the underlying deterministic FEM simulations. These inconsistencies are more noticeable when sampling at high rates and reduce discriminatory performance. In addition, the mutual information captured at lower sampling rates are likely to be positively biased because the start of the impacts were aligned with 0.2 ms precision provided by the native sampling rate of the sensor. These issues may be resolved using advanced learning algorithms that are adaptable to the characteristics of sensors and robust to experimental noise.

One of the goals of this work was to highlight the advantages of coding tactile stimuli through spatiotemporal representations, which FA-1 afferents are well suited for. Although FA-1 like representations have been demonstrated to work well under dynamic conditions, we do not diminish the importance of other mechanoreceptors; namely, the SA-1, FA-II, and SA-II, and the value of accurate pressure measurements. Indeed, an efficient processing strategy could be implemented where the saliency of tactile signals are characterized rapidly based on the temporal structure to prime higher order attentional mechanisms. Processing resources can then be allocated to analyze the stimulus in greater detail as spikes from SA type mechanoreceptors are accumulated over time.

Beyond the tactile domain, neuromorphic event-based sensory processing has been implemented in vision and audition (Lichtsteiner et al., 2008; Posch et al., 2011; Brandli et al., 2014; Liu et al., 2014). Research in spike based processing in these fields are more established, partly due to the availability of silicon retinae and cochleas (Chan et al., 2007; Delbrück et al., 2010). Dedicated processing hardware and algorithms have also been developed to interface with spatiotemporal features captured by such sensors (Liu and Delbrück, 2010). Due to its distributed nature and challenging requirements to be mechanically flexible and stretchable, building an inherently event-based tactile sensor array is significantly more challenging. Recent progress in material science and manufacturing technology have opened new opportunities such as using organic transistors to create digital mechanoreceptors (Tee et al., 2015). Such innovative components and applications will hopefully increase interests and development in tactile sensing.

## Author contributions

WL conceptualized the idea, conducted the experiments, analyzed the results and wrote the manuscript. NT directed the research and NT and SK helped to interpret the results. SK and NT revised the manuscript.

## Funding

This work is supported by a NPRP grant from the Qatar National Research Fund under the grant No. NPRP 7-673-2-251. The statements made herein are solely the responsibility of the authors.

### Conflict of interest statement

The authors declare that the research was conducted in the absence of any commercial or financial relationships that could be construed as a potential conflict of interest.
